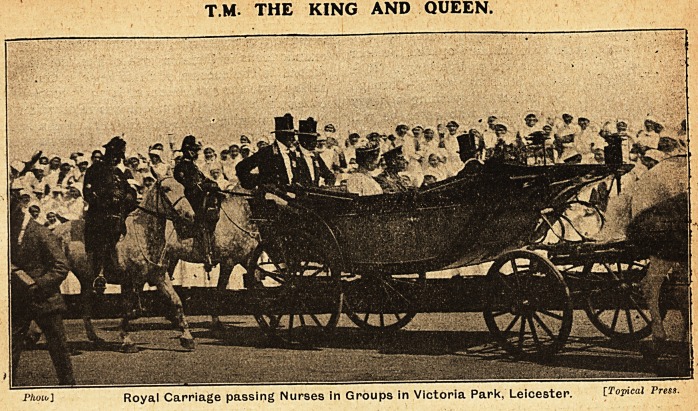# The Importance of a Low Registration Fee

**Published:** 1919-06-21

**Authors:** 


### T.M. THE KING AND QUEEN.

Photo] Royal Carriage passing Nurses in Groups in Victoria Park, Leicester. [Topical Press.

### THE MATRONS' AND SISTERS' DEPARTMENT.

### THE IMPORTANCE OF A LOW REGISTRATION FEE.

THE value of State registration must depend on the extent to which it commands unanimity from the nurses for whose benefit it is secured. It is not a compulsory measure. Although it is designed for the protection both of nurses and the public, it has to be remembered from the outset that there are forces at work tending to induce both nurses and the public to reject its protection.

We are not at the moment concerned with the "years of grace" during which women who come short of the minimum modern standard of training will be permitted, indeed encouraged, to register. Nurses who have been some years at work realise the value of the protection offered to them by registration and eagerly demand it. But what after all can the newly-fledged nurse, just emerging from the status of a probationer, know about the conditions to which she will be exposed when she begins work? Yet State registration in order to become thoroughly successful must command the adhesion of thousands of these budding nurses every year. It must present itself to the probationer at the conclusion of her course as the indispensable passport for obtaining the fruits of her labours. There is need to be very wary lest by raising too clumsy a barrier before this professional gate we induce young nurses to slink round another way in defiance of common sense, and under a delusion as to their true interests. It would be particularly foolish to undervalue this danger. America has given us an object-lesson in the extent to which State registration may fall short of its purposes just because newly trained nurses cannot be brought to understand its value.

To impose a fee of three or four guineas on a nurse for examination and registration just at her poorest moment is to hit her in a very tender spot. She dislikes the idea of another examination excessively, having just "graduated," as the phrase goes, in her own training-school. If it were offered to her free of cost she would be far better disposed towards it as the passport to future success and the condition on which she was permitted to inscribe her name on the register at the nominal fee of one guinea. But so surely as we attempt to make her pay out of her slender income the whole cost of the registrar, the examiners, the holding of examinations, the issue of the register, we shall find that her conviction of its value to her personally is too weak to bring the average nurse to the point. True, she will be strongly urged thereto by her superiors, the sisters and the matron. But she may quite possibly imagine they are not entirely disinterested. She may argue that she is not proposing to be a sister or a matron, and that she has already a promise of good work, whether she be registered or not; she will plead that she cannot afford it now, but may do so later on. In short, she might wriggle out of the examination with its demand for payment of high fees, and in so doing she weakens the whole fabric of registration, together with the solidity of the entire profession, about which she knows little and might care less.

**Importance of a Low Registration Fee—(*continued*).**

The responsibility of the shepherd does not end when he has prepared the fold and opened the door to it. In fact it is redoubled at this stage. It is now his duty to see that the sheep are got through the opening. If he be seen flourishing a heavy stick over the backs of those who enter he will not increase the desire of the sheep to get in. And be it well understood that it is fully as much to the advantage of the shepherd as to the sheep that they should all be folded. Quite possibly a group of sheep might do well enough outside the fold, and secure a patch of pasture from which the others for good reasons were cut off. But the outsiders are exposed to dangers they wot not of, and the shepherd thereby to loss of status. In fact the credit and safety of the flock which refuses to let itself be folded is seriously endangered.

A strong College of nursing sufficiently endowed to provide the cost of regulating the entrance to the profession without appealing for aid to young women imperfectly acquainted with the uses of entrance examinations is therefore one of the essential conditions to the success of State registration. Once realise that the profession at large is more deeply concerned with getting unanimous registration than any one member of the nursing service can be, and the importance of keeping the fee as low as possible is plainly evident.

The defect of all registration schemes hitherto has been that they have been built on the easy theory that all nurses will at once perceive it to be advantageous to be registered. This theory has been disproved in every country where voluntary State registration has been introduced. There are too many people industriously bent on exploiting nurses for plausible reasons to be wanting for not registering. To impose a high registration and examination fee is to play into their hands.

## Figures and Tables

**Figure f1:**